# Response of macroinvertebrate communities to land use and water quality in Wudalianchi Lake

**DOI:** 10.1002/ece3.7140

**Published:** 2021-01-07

**Authors:** Xue Du, Dan Song, Kun Ming, Xing Jin, Huibo Wang, Le Wang, Hui Liu, Chen Zhao, Tangbin Huo

**Affiliations:** ^1^ Heilongjiang River Fishery Research Institute Chinese Academy of Fishery Sciences Harbin China; ^2^ Harbin Management Station of the Forth Administration Bureau of Reserve assets The Joint Logistics Support Force of PLA Harbin China

**Keywords:** buffer zone, land use types, macroinvertebrate, water quality

## Abstract

Macroinvertebrate assemblages are structured by a number of abiotic and biotic factors interacting simultaneously. We investigated macroinvertebrate assemblages along gradients of human disturbance and morphometric characteristics in five lakes connected by the same stream. We aimed to assess the relative effects of environmental gradients on macroinvertebrate assemblages and to investigate whether water quality effects on the assemblages were correlated with buffer land use. There were significant differences in macroinvertebrate community compositions among lakes, and our results indicated that oligochaetes (mainly *Limnodrilu*s) and insects (mainly *Chironomus*) contributed highly to the differences. We used redundancy analysis with variation partitioning to quantify the independent and combined anthropogenic effects of water quality and land use gradients on the macroinvertebrate community. The independent effect of water quality was responsible for 17% of the total variance in macroinvertebrate community composition, the independent effect of buffer land use accounted for 6% of variation, and the combined variation between land use change and water quality accounted for 12%. Our study indicated that both the independent effects of land use and within‐lake water quality can explain the influence in macroinvertebrate assemblages, with significant interactions between the two. This is rather important to notice that changes in buffer land use generally may alter nutrient inputs and thus severely affect abiotic conditions encountered by macroinvertebrate. Our study demonstrates that considering buffer zone effects explicitly may be significant in the selection and application of conservation and management strategies.

## Graphical abstract

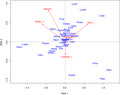

## 1 INTRODUCTION

Understanding the responses of species and communities to natural and human‐induced environmental changes of lakes has been a major focus of ecology. A multitude of natural and anthropogenic factors interacts simultaneously on lake ecosystems resulting in additive, antagonistic, or synergistic effects (Jackson et al., 2016; McGoff et al., 2013). Much ecological research is increasingly driven by the need to quantify the single and combined effects of multiple stressors on freshwater aquatic assemblages (Jackson et al., 2016; Ormerod et al., 2010). The effects of anthropogenic land use changes have been acknowledged as one of the most important drivers of degradation of lake ecosystems. Indeed, physicochemical and biological characteristics of lakes are strongly correlated with surrounding land use types and changes in land use such as increased percentage of agriculture and urbanization that are common causes of lake nutrient enrichment, habitat degradation (Meng et al., 2018; Nielsen et al., 2012), and biodiversity loss (Meier et al., 2015; Twardochleb & Olden, 2016).

Wudalianchi Lake (48° 40' N ~ 48° 47' N, 126° 06' E ~ 126° 15' E), the second largest volcanic lava barrier lake in China, is located in Wudalianchi City, a county‐level city under the administration of Heihe City in Heilongjiang Province. The lava flows produced by the last eruption of volcanoes occurred in around 1,720 resulted in the blockage of the Shilong River, separating the channel into five consecutive lakes: the Lotus Lake, Yanshan Mountain Lake, White Dragon Lake, Crane‐chirping Lake, and Ruyi Lake (Jing et al., 2014), where have been developed as tourist attractions. In the past several decades, enhanced anthropogenic activities within the watershed, including urbanization, tourism, and deforestation for agricultural development, have deteriorated the lake environment (Gui et al., 2012). Within this context, evaluating anthropogenic impacts on lake functionality is an essential step for the selection and application of conservation and management strategies.

Macroinvertebrates are one component of food webs in freshwater ecosystems, constituting an important link between primary producers, detrital deposits and higher trophic levels, and they are vital to the functioning of lakes, contributing to nutrient cycling and decomposition (Wallace & Webster, 1996). Macroinvertebrates respond to nutrient enrichments, turbidity, changes in sediment composition, and morphological alterations, often originating from anthropogenic activities (Meng et al., 2018; Porst et al., 2019). Nutrient enrichment alters the community composition of lake macroinvertebrates (Brauns et al., 2007) and homogenizes assemblages of littoral macroinvertebrates (Donohue et al., 2009). Water quality of lakes is integrally linked to land use properties. Lake water quality can be directly influenced by surrounding land uses through receiving materials from landscapes and thus macroinvertebrate assemblages (McGoff et al., 2013; Vanni et al., 2011) and indirectly through morphological alteration of lakeshores (Brauns et al., 2007). For instance, inputs of nutrients (mainly nitrogen and phosphorus) from urban and agricultural substantially result in lake eutrophication (Carpenter et al., 1998), which might have caused severe environmental deterioration and diversity loss of macroinvertebrate assemblages (Cuffney et al., 2010). Lake buffer land use characteristics play a crucial role in potential nutrient loading to lakes. Many studies have demonstrated that buffer land use may be more important in influencing the presence of macroinvertebrate species (Brauns et al., 2007; Pilotto et al., 2015) or species diversity (McGoff et al., 2013).

In this study, we investigated the macroinvertebrate assemblages of the five lakes and their relationships with morphometric variables and environmental pressure gradients. We chose these five lakes because they are in the same watershed connected by the same stream, but differ in lake morphology (i.e., surface area, maximum depth and perimeter) and human‐induced environmental changes of lakes (land use types in the buffer zone of 100 m width around lakes and water quality), which may result in different abiotic–biotic associations. We aimed to assess the relative effects of environmental gradient factors on macroinvertebrate assemblages and to investigate whether water quality effects on macroinvertebrate assemblages were correlated with buffer land use. We hypothesized that buffer land use and lake water quality are strongly coupled and their joint effects could explain substantial amounts of variation in macroinvertebrate communities.

## 2 MATERIALS AND METHODS

### 2.1 Study area

**Figure 1:**
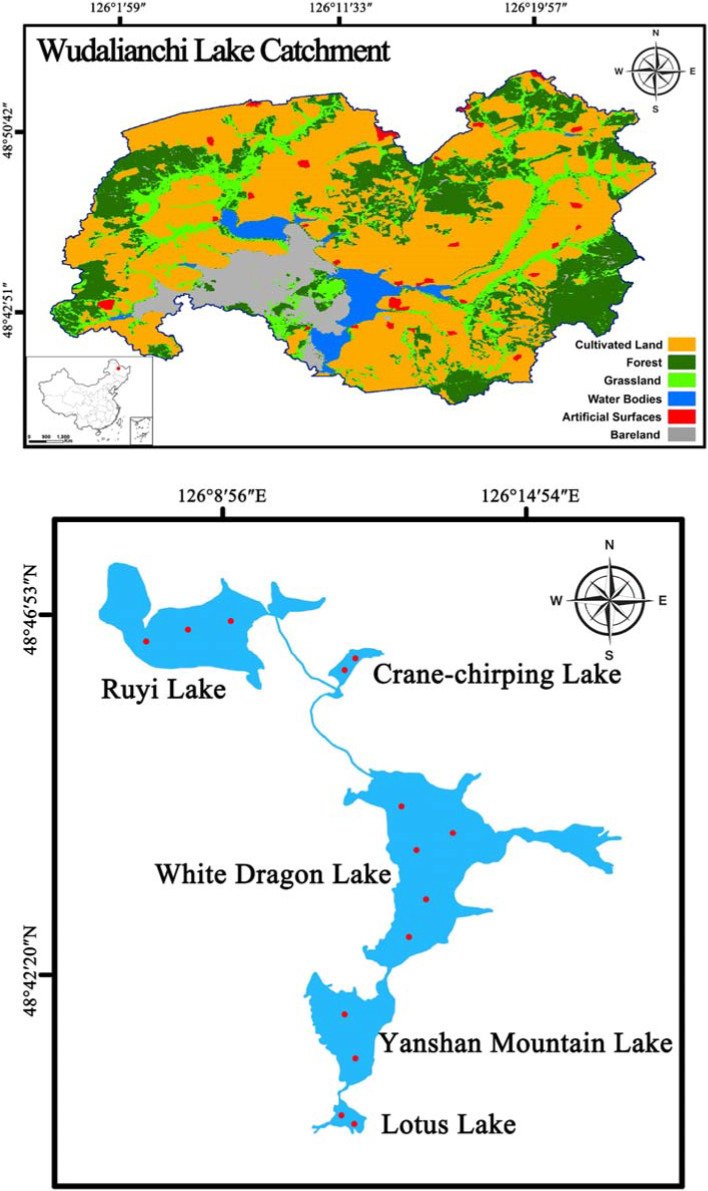
Map of Wudalianchi Lake Catchment in China (up) showing the sampling sites (below)

The study took place in Wudalianchi Lake (Figure 1). The lakes were created together by volcano eruption and composed of five lakes that are in the same watershed connected by the same stream (Figure 1). The five lakes were selected for study based on diverse representation of morphometric characteristics. The five lakes ranged in surface area from 0.43 to 10.58 km^2^ and had a maximum depth from 4 to 12 m (Table 1). Moreover, the five lakes vary in land use. The Ruyi Lake is surrounded by agricultural land and is prone to receiving terrestrial material input, while White Dragon Lake is close to Wudalianchi City, a city famous for tourism in China (Gui et al., 2012).

**Table 1:** Description of morphometric and environmental factors and macroinvertebrates from fiver lakes of Wudalianchi Lake; mean values are indicated for quantitative variables

Variables	Units	Lotus Lake	Yanshan Mountain Lake	White Dragon Lake	Crane‐chirping Lake	Ruyi Lake
Morphometric variables						
Surface area	km^2^	0.43	3.37	10.58	0.48	5.88
Perimeter	km	4.26	14.48	39.43	4.48	21.89
Maximum depth	m	4	9.1	12	5	6
Buffer land use						
Agricultural land use	%	47.3	48.5	51.1	54.2	52.3
Artificial land use	%	2.7	2.8	4.3	3.7	2.6
Forests	%	30.8	30.5	26.3	17.9	18.3
Water quality						
Water temperature	°C	17.2	17.1	16.7	15.8	15.7
Secchi depth	m	0.63	1.42	1.09	0.88	0.91
Dissolved oxygen	mg/l	9.3	9.7	10.3	7.5	8.6
Total phosphorus	mg/l	0.31	0.32	0.34	0.21	0.61
Total nitrogen	mg/l	1.11	0.97	1.15	1.30	1.57
Chlorophyll *a*	μg/l	16.7	15.5	17.8	18.4	20.5
Macroinvertebrates						
Abundance	ind./m^2^	49.99	191.55	226.79	46.86	1,122.78
Species richness		7	24	27	7	26

### 2.2 Macroinvertebrate data

Benthic macroinvertebrates were collected in April, July, and October 2017, respectively (Figure 1). Sampling sites were selected based on the area of the lake, with 2 to 5 sites chosen for each lake. The substrata of these sampling sites were mainly characterized by muddy sediment. Three replicate samples were taken at all sampling sites of lakes using a modified Peterson grab sampler (mean sampling area: 0.0625 m^2^) and sieved in situ through a nylon net with a mesh size of 300 μm. Macroinvertebrates were manually sorted out from the materials retained from the 300 μm sieve and preserved in 80% alcohol in plastic jars in the field. Densities in number of individuals per m^2^ were averaged among the samples. In the laboratory, all individuals sampled were identified to the lowest feasible taxonomic level, generally species or genus (Liu et al., 1979; Morse et al., 1994; Tang, 2006; Wang, 2002).

### 2.3 Explanatory data

The lake morphological variables consisted of lake surface area (km^2^), perimeter (km), and maximum depth (Table 1). Lake surface area and perimeter were calculated using ArcGIS (ver. 10.7). Maximum depth (m) was obtained from *Encyclopedia of Rivers and Lakes in China Section of Heilongjiang River and Liaohe River* (Jing et al., 2014).

Buffer zone variables were calculated using ArcGIS to obtain the percentage of different land use types. We used buffer land use in the proximity of the lakes as an indicator of human‐induced morphological alteration (McGoff et al., 2013; Pilotto et al., 2015). We estimated the land use within a 100 m radius around each lake. The land use variables comprised proportions of forests, agricultural, and artificial areas.

We measured site‐level physicochemical variables at the time of macroinvertebrate sampling. We determined dissolved oxygen and water temperature with a portable YSI Professional Plus instrument (YSI Incorporated, Yellow Springs). Secchi depth (m) was measured at the same locations as macroinvertebrate samples in the field. Water depth was measured with a handheld depth finder. Water samples for chemical analysis, including dissolved oxygen (mg/L), total phosphorus (mg/L), total nitrogen (mg/L), and chlorophyll *a* concentration (mg/L), were directly collected into 5‐L polypropylene bottles at a depth of 0.5 m and were kept cool and immediately transport to the laboratory. Total phosphorus and total nitrogen concentrations were measured with ascorbic acid method and cadmium reduction method, respectively, following persulfate digestion (APHA, 1992). Water samples for determining chlorophyll *a* concentrations were filtered through Whatman GF/F filters and determined fluorometrically after methanol extraction (Yentsch & Menzel, 1963).

### 2.4 Statistical analysis

Before data analyses, we arcsine transformed the land use, and log‐transformed morphometric and water quality variables with skewness greater than one, while we Hellinger‐transformed macroinvertebrate abundance to better approximate normal distribution (Legendre & Legendre, 2012).

We investigated variation in physicochemical and morphological characteristics and taxonomic composition of macroinvertebrate assemblages of the five lakes using principal component analysis (PCA). Then, differences in macroinvertebrate community structure among the lakes were tested using one‐way analysis of similarities (ANOSIM; Clarke, 1993). SIMPER analysis was used to identify the contribution of each species to the average similarity within lake (Clarke & Warwick, 2001).

To reduce the dimensionality and collinearity of the dataset and identify major environmental gradients, we used the axes of principal component analyses (PCA) of three groups of environmental gradients: lake morphology (Morph_1), land use (Landuse_1 and Landuse_2), and physicochemical variables (WQ_1 and Depth_1) (see Appendix S1 in Figs S1–S3). We were interested in the effects of environmental gradients in structuring the macroinvertebrate community, so redundancy analysis (RDA) was used to determine the effects of lake morphology, land use, and physicochemical variables on macroinvertebrate community structure in five lakes. As we hypothesized that buffer land use and lake water quality are strongly coupled and their joint effects could explain substantial amounts of variation in macroinvertebrate communities, we also used variance‐partitioning (VP) analysis (Legendre & Legendre, 2012) to determine how much of the variation of macroinvertebrate species occurrence is explained by independent anthropogenic disturbance measurements, that is, buffer land use (Landuse_1 and Landuse_2) and physicochemical variables (WQ_1), as well as their combined effects and unexplained variance. All analyses were obtained in the vegan package (Oksanen et al., 2019) of the R (version 3.6.3) statistical software (R Core Team, 2020).

## 3 RESULTS

**Figure 2:**
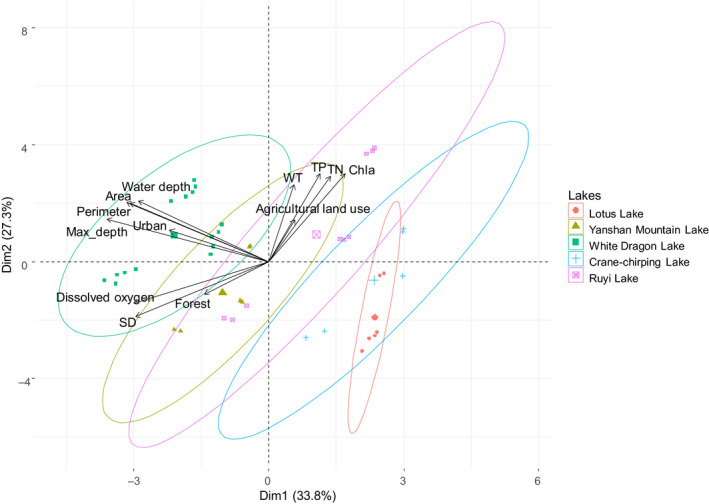
Principal components analysis (PCA) of the environmental gradient variables included in the study and ordination of the sites. Single lakes are represented by ellipses

The PCA (Figure 2) for the three groups explanatory variables showed that axes 1 and 2 explained a variance of 61.1%. Axis 1 explained 33.8% of the total variance with lake morphological variables, dissolved oxygen, Secchi depth, and urban land use showing a negative loading. This axis fundamentally distinguished two small lakes, that is, the Lotus and Crane‐chirping Lake, from other three relatively larger lakes. Moreover, the largest and deepest lake, the White Dragon Lake, has a larger buffer urban area. Axis 2 explained 27.3% of the total variance with total phosphorus, total nitrogen, chlorophyll *a*, and agricultural land use showing a positive loading. Axis 2 did not distinguish among lakes, indicating a positive correlation between nutrients and agricultural land use and the five lakes.

Overall, 44 macroinvertebrate taxa were collected from the five lakes during the three seasons of sampling, including 26 aquatic insects, eight gastropods, five oligochaetes, three leeches, and two bivalves (Table S1). Total macroinvertebrate abundance varied greatly between 26.5 ind./m^2^ and 1,135.78 ind./m^2^, with high values being recorded in Ruyi Lake. In general, these lakes were mainly dominated by pollution‐tolerant oligochaetes and chironomids (Table S1). *Limnodrilus hoffmeisteri* was the dominant oligochaete in the five lakes, while *Chironomus dorsalis* was the dominant chironomid. Species richness was highest in the White Dragon Lake (*n* = 27), followed by the Ruyi Lake (*n* = 26) and Yanshan Mountain Lake (*n* = 24), and lowest in the Lotus and Crane‐chirping Lake (Table 1).

**Figure 3:**
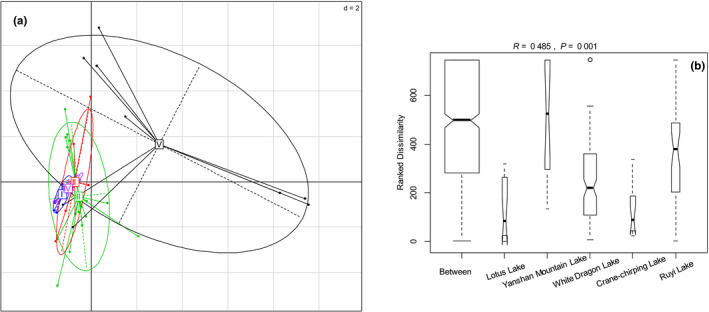
The variation of Hellinger‐transformed macroinvertebrate (a) taxonomic composition among lakes based on principal component analyses (PCA). Single lakes are represented by ellipses. I = Lotus Lake; II = Yanshan Mountain Lake; III = White Dragon Lake; IV = Crane‐chirping Lake; V = Ruyi Lake. Analysis of similarities (ANOSIM) boxplot displaying the differences in the macroinvertebrate (b) taxonomic composition between and within single lakes

Variation in macroinvertebrate community composition among lakes was pronounced, although community compositions displayed an overlap among lakes in the PCA plot (Figure 3a). The result of ANOSIM (Figure 3b) also indicated that there were significant differences in macroinvertebrate community compositions among lakes (*R* = 0.485, *p* = .001). The SIMPER analysis indicated that *Phalera flavescens* and *Chironomus kiiensis* characterized Ruyi Lake (Table 2). Except Lotus Lake, insect *C. dorsalis* and oligochaete *L. hoffmeisteri* occurred widely in the other four lakes, and species belonging to families Tubificidae and Chironomidae contributed to differences among these lakes (Table 2). *C. sinicus* and *C. kiiensis* were the main contributing insects of Yanshan Mountain and Crane‐chirping Lake, respectively, while oligochaetes *Tubifex tubifex and L. amblysetus* were generally more abundant in White Dragon and Ruyi Lake, respectively.

**Table 2:** Similarity of percentage (SIMPER) analysis of macroinvertebrate community composition in Wudalianchi Lake. Values represent the percentage contribution (%) of each species to the within‐lake similarity (only the most important species contributing globally up to 90% of the within‐lake similarity are shown)

Species	Lotus Lake	Yanshan Mountain Lake	White Dragon Lake	Crane‐chirping Lake	Ruyi Lake
*Phalera flavescens*	52.71	5.01		9.46	
*Chironomus kiiensis*	28.89			18.92	7.09
*Viviparus chui*	7.83			12.3	
*Limnodrilus claparedianus*	4.65	7.46	9.97		2.71
*Chironomus dorsalis*		27.22	18.9	23.61	20.58
*Limnodrilus hoffmeisteri*		19.98	37.23	16.89	7.88
*Chironomus sinicus*		15.63			
*Tanypus vilipennis*		3.58	3.36		6.96
*Bellamya aeruginosa*		2.92			
*Tubifex tubifex*		2.76	17.3		5.61
*Propsilocerus akamusi*		2.58			
*Tanypus chinensis*		2.41			
*Limnodrilus amblysetus*		2.37			11.8
*Limnodrilus udekemianus*			5.16		3.25
*Chaoborus* sp.				12.21	2.97
*Whitmania* sp.					10.11
*Ephemera shengmi*					5.09
*Diplocladius* sp.					2.3
*Helobdella stagnalis*					2.05
*Pentaneura* sp.					1.97

**Figure 4:**
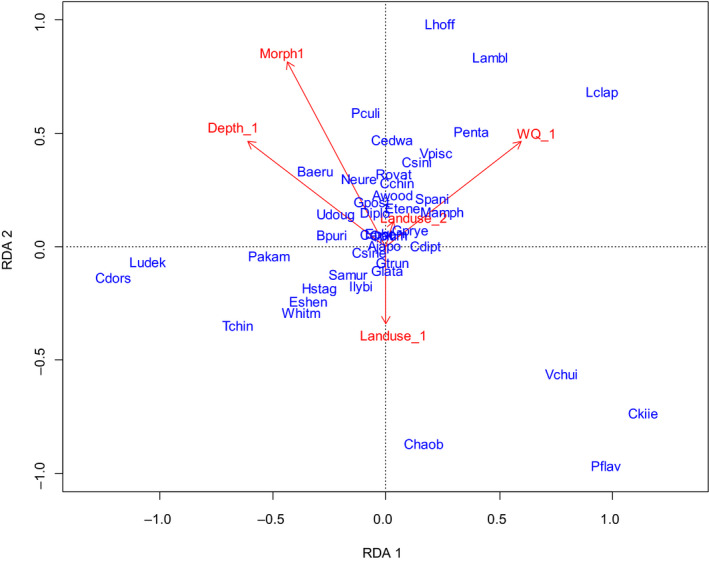
Redundancy analysis (RDA) predicting macroinvertebrate species composition by selected environmental gradient components

Results of redundancy analysis (RDA) for macroinvertebrate assemblages (Figure 4) showed that the first axis represented a gradient of water quality explained 42.2% of the variance in the species data, while the second axis explained 29.4% of the variance in the species data and represented a gradient of lake morphology. Among the strongest species–environment associations, we found that oligochaetes such as *L. hoffmeisteri*, *L. claparedianus,* and *L. amblysetus* were positively associated with physicochemical variables (Figure 4). Insects such as *C. kiiensis* and *Chaoborus* sp. were positively associated with physicochemical variables and land use (Figure 4).

**Figure 5:**
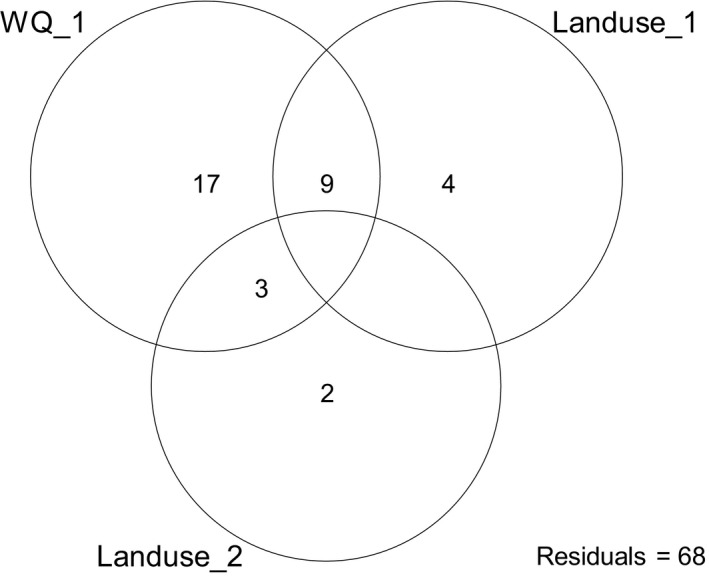
Variation partitioning of macroinvertebrate species composition explained by environmental predictors

Variation partitioning result showed that water quality and land use explained 32% of the variation in macroinvertebrate assemblages (Figure 5). The main independent explanatory group was water quality among lakes, which accounted for 17% of total variance. The independent effect of Landuse_1 that represented agricultural land use and Landuse_2 that represented artificial land use accounted for 4 and 2% of total variance, respectively. The joint effects of buffer land use and water quality explained 12% of the variation in macroinvertebrate assemblages.

## 4 DISCUSSION

We examined three sources of variation in macroinvertebrate community, namely lake morphology, buffer land use, and physicochemical conditions. Our results highlighted the importance of within‐lake water quality and buffer land use types as potential drivers shaping macroinvertebrate community structure. We also observed that the joint effects of buffer land use and water quality were significant determinants of community structure.

We expected that within‐lake physicochemical conditions would explain substantial amounts of variation in macroinvertebrate communities. We found clear support for this expectation from the results of RDA and VP. Nutrient concentrations and chlorophyll *a* were the variables most strongly related to the PCA water quality gradient (WQ_1) at the site and lake scales (Fig. S3). Although there were significant differences in macroinvertebrate composition among lakes, our results indicated that particular taxa contributed highly to the similarities among lakes. In this study, Wudalianchi Lake was dominated by oligochaetes (mainly *Limnodrilus*) and insects (mainly *Chironomus*) and these species were found to be strongly associated with physicochemical variables. There are several mechanisms potentially explaining this observation. Our study lakes are in the same watershed connected by the same stream; therefore, the actively dispersing organisms (i.e., macroinvertebrates) may overcome lake boundaries more easily via watercourses (De Bie et al., 2012). The dominant oligochaete species were often associated with eutrophic lakes in China (Cai et al., 2017; Li et al., 2016), while chironomids often reach high densities in productive waters (Corbi & Trivinho‐Strixino, 2017; Gezie et al., 2017; Vaughan et al., 2008). In the present study, *L. hoffmeisteri* and *C. dorsalis* were the most abundant species and maintained high similarity in the five lakes, indicating their ubiquitous distribution as habitat generalists and their role as indicators of eutrophication and anthropogenic alteration of Wudalianchi Lake.

Land use was recognized as one of the most important anthropogenic drivers of macroinvertebrate species composition for freshwater lakes. The observed buffer land use effect on macroinvertebrate communities concurs with several previous studies. Pilotto et al. (2015) found significant effects of land use within a 200 m zone from the lake edge on littoral macroinvertebrates and attributed this to morphological alterations to shorelines. McGoff and Sandin (2012) revealed that macroinvertebrate community composition significantly responded to changes in land use at a 50 m subcorridor scale in Swedish lakes. Similarly, Johnson et al. (2004) indicated that riparian buffer land use had a larger effect on functional feeding guild composition of macroinvertebrates. Evidence from recent studies performed at the lake buffer zone demonstrated that macroinvertebrate occurrence and diversity is largely affected by environmental heterogeneity (Schmalz et al., 2015). Agriculture and urban land uses have been shown to degrade macroinvertebrate assemblage habitats by excessive nutrient inputs to lakes ecosystems (Carpenter et al., 1998) that can result in water deterioration, such as decreased water transparency and oxygen, and toxic algal blooms, or reducing habitat heterogeneity and available surface area (Brauns et al., 2007).

The notable influence of combined variances of land use and water quality on macroinvertebrate suggests that environmental variables at the buffer zones play critical roles in influencing macroinvertebrate. Land use and water quality changes occur concomitantly (Castro et al., 2018; Ferreira et al., 2017); in our study, increasing agriculture land use was found to be strongly correlated with increasing nutrients, indicating the importance of the buffer land use for within‐lake water quality. Furthermore, land use next to the lake shoreline has the potential to directly alter the morphology of the littoral zone and, thus, alter the habitat of aquatic macroinvertebrate (McGoff et al., 2013). For example, macroinvertebrate composition differed among shore types and taxon richness of macroinvertebrate decreased with morphological alterations across European lakes (Porst et al., 2019). According to the German Working Group on water issues, agricultural land use types exceeding a 40% of the catchment area are considered as important morphological alterations and pressures on freshwaters water quality (LAWA, 2003). For Wudalianchi Lake, agricultural land use types exceeding a 40% of the total buffer area, thus revealing a stronger habitat alteration and buffer effects on water quality. Moreover, the five lakes have relatively lotic water and low dynamic ratios [sqrt(surface area) /mean depth = 0.17 ~ 0.48] (Håkanson, 1982), which particularly enables accumulation of nutrients. As a famous tourist attraction, Wudalianchi is being impacted by ongoing development on and around lakeshores including residential and commercial development, road construction, with assemblage structure responding to habitat alteration.

Our study also showed that lake morphometry plays a significant role in modulating variations in macroinvertebrate communities. Morphometry is a static variable over timescales of thousands to millions of years that cannot be significantly influenced by humans (Walters et al., 2009). The significance of lake morphometric variables as predictors in macroinvertebrate community was useful for improving the predictive capabilities of models. Lake size has been shown to impact macroinvertebrate assemblages by increasing habitat heterogeneity and available surface area (McGoff et al., 2013). Relatively higher macroinvertebrate species richness was positively correlated with larger lakes (Brown, 1985). In our study, species richness was higher in relatively larger lake, agreeing with previous studies, attributable to higher variability in available habitats (Hoffmann & Dodson, 2005).

## 5 CONCLUSION

In conclusion, macroinvertebrate communities were strongly associated with anthropogenic pressures in lakes. Our study indicates that both the land use, and within‐lake water quality can solely explain the influence in macroinvertebrate assemblages, with significant interactions between the two. This is rather important to notice that changes in buffer land use generally may alter nutrient inputs and thus severely affect abiotic conditions encountered by macroinvertebrate. Our study demonstrates that considering buffer zone effects explicitly may be significant in the selection and application of conservation and management strategies.

## CONFLICT OF INTEREST

The authors declare no conflict of interest.

## AUTHOR CONTRIBUTIONS

**Xue Du:** Conceptualization (equal); field sampling (equal); formal analysis (equal); funding acquisition (equal); methodology (equal); supervision (equal); visualization (equal); writing‐original draft (lead). **Dan Song:** Conceptualization (equal); formal analysis (equal); methodology (equal); writing‐review and editing (equal). **Kun Ming:** Field sampling (equal); methodology (equal); writing‐review and editing (equal). **Xing Jin:** Funding acquisition (equal); supervision (equal); writing‐review and editing (equal). **Huibo Wang:** Field sampling (equal); writing‐review and editing (equal). **Le Wang:** Field sampling (equal); writing‐review and editing (equal). Hui Liu: Field sampling (equal); writing‐review and editing (equal). **Chen Zhao:** Writing‐review and editing (equal). **Tangbin Huo:** Conceptualization (equal); field sampling (equal); funding acquisition (equal); writing‐review and editing (equal); methodology (equal); supervision (equal).

## Supporting information

Appendix S1

## Data Availability

Macroinvertebrate and environmental variable data were deposited in the Dryad Digital Repository, https://doi.org/10.5061/dryad.qnk98sff8. Other relevant data were sourced from publicly accessible repositories or manuscripts.
