# Supplementary material for: Response of macroinvertebrate communities to land use and water quality in Wudalianchi Lake
Source: Ecol Evol. 2021 Jan 7;11(3):1368–77. doi: 10.1002/ece3.7140 (PMC7863386; doi:10.1002/ece3.7140)
Supplement: Supplementary file 1 — Appendix S1 [file ECE3-11-1368-s001.docx]

**Appendix S1.** Derivation of environmental gradients

Three groups of environmental gradients were identified, i.e. lake morphology, land use, and physicochemical variables. As these variables were strongly intercorrelated, different principal component analysis (PCA) for each environmental gradient was carried out. According to the Kaiser-Guttman criterion (Legendre, & Legendre, 2012), only axes with eigenvalues > 1.0 were retained for interpretation.

PCA of lake morphological variables accounted for 95.3% of the total variation for the first axis (Morph_1). This axis reflected that large lakes with increasing perimeter and maximum depth (Fig. S1). Two axes were retained from the PCA of land use variables, which explained 98.2% of the total variance (Fig. S2). The first axis (Landuse_1) was positively correlated with agricultural land use, while the second axis (Landuse_2) was positively correlated with urban areas. Two axes were retained from the PCA of physicochemical variables, which explained 77.5% of the variance (Fig. S3). The first axis (WQ_1) explained 51.4% and was positively correlated with secchi depth and dissolved oxygen but negatively correlated to chlorophyll *a*, total phosphorus, total nitrogen and water temperature. We reversed the sign of WQ_1 scores, so that positive scores indicated increased deterioration in water quality. The second axis (Depth_1) explained 26.1% of the variance and was positively correlated to water depth.

**FIGURE S1** Principal component analysis of lake morphological variables.

**FIGURE S2** Principal component analysis of buffer land use variables.

**FIGURE S3** Principal component analysis of physicochemical condition variables.

**TABLE S1** Mean abundance (ind./m^2^) for the 44 macroinvertebrate species collected in each lake of the Wudalianchi Lake

| Phylum | Class | Family | Species | Species abbreviation | Lotus Lake | Yanshan Mountain Lake | White Dragon Lake | Crane-chirping Lake | Ruyi Lake |
| --- | --- | --- | --- | --- | --- | --- | --- | --- | --- |
|  |  |  |  |  |  |  |  |  |  |
| Annelida | Clitellata | Hirudinidae | *Whitmania* sp. | Whitm |  | 6.22 |  |  | 17 |
| Annelida | Hirudinea | Glossiphoniidae | *Glossiphonia lata* | Glata |  | 8.89 |  |  |  |
| Annelida | Hirudinea | Glossiphoniidae | *Helobdella stagnalis* | Hstag |  | 0.18 |  |  | 5.33 |
| Annelida | Oligochaeta | Tubificidae | *Limnodrilus amblysetus* | Lambl |  | 3.78 | 14.7 |  | 128 |
| Annelida | Oligochaeta | Tubificidae | *Limnodrilus claparedianus* | Lclap | 20.61 | 4.84 | 23.57 | 2.6 | 130 |
| Annelida | Oligochaeta | Tubificidae | *Limnodrilus hoffmeisteri* | Lhoff | 10.22 | 13.04 | 42.66 | 1.08 | 82.56 |
| Annelida | Oligochaeta | Tubificidae | *Limnodrilus udekemianus* | Ludek |  | 5 | 28.05 |  | 22.67 |
| Annelida | Oligochaeta | Tubificidae | *Tubifex tubifex* | Ttubi | 31.5 | 3.56 | 36.85 |  | 97.89 |
| Arthropoda | Insecta | Baetidae | *Cloeon dipterum* | Cdipt |  | 0.88 |  |  |  |
| Arthropoda | Insecta | Belostomatidae | *Appasus japonicus* | Ajapo |  | 3 |  |  |  |
| Arthropoda | Insecta | Caenidae | *Caenis sinensis* | Csine |  | 1.03 |  |  |  |
| Arthropoda | Insecta | Ceratopogonidae | Ceratopogonidae sp. | Cerat |  |  |  |  | 3.22 |
| Arthropoda | Insecta | Chaoboridae | *Chaoborus* sp. | Chaob | 2.5 | 0.33 |  | 12.17 | 26.67 |
| Arthropoda | Insecta | Chironomidae | *Chironomus dorsalis* | Cdors |  | 18.67 | 30.83 | 8.67 | 170.67 |
| Arthropoda | Insecta | Chironomidae | *Chironomus kiiensis* | Ckiie | 2.33 | 2.13 | 2.4 | 4.67 | 148.44 |
| Arthropoda | Insecta | Chironomidae | *Chironomus plumosus* | Cplum |  |  | 1.07 |  |  |
| Arthropoda | Insecta | Chironomidae | *Chironomus sinicus* | Csini |  | 81.07 | 2.4 |  | 35.56 |
| Arthropoda | Insecta | Chironomidae | *Cladopelma edwardsi* | Cedwa |  |  | 0.27 |  | 10.33 |
| Arthropoda | Insecta | Chironomidae | *Diplocladius* sp. | Diplo |  |  |  |  | 30 |
| Arthropoda | Insecta | Chironomidae | *Pentaneura* sp. | Penta |  | 10.67 | 8.53 |  |  |
| Arthropoda | Insecta | Chironomidae | *Procladius culiciformis* | Pculi |  | 1.93 | 2.4 |  | 1.78 |
| Arthropoda | Insecta | Chironomidae | *Propsilocerus akamusi* | Pakam |  | 6.53 | 2.88 |  | 0.33 |
| Arthropoda | Insecta | Chironomidae | *Spaniotoma* sp. | Spani |  | 0.33 | 3.2 |  |  |
| Arthropoda | Insecta | Chironomidae | *Tanypus chinensis* | Tchin |  | 7.4 | 6.97 |  |  |
| Arthropoda | Insecta | Chironomidae | *Tanypus vilipennis* | Tvili |  | 5.07 | 7.73 |  | 167.78 |
| Arthropoda | Insecta | Dytiscidae | *Ilybius* sp. | Ilybi |  |  |  |  | 1.22 |
| Arthropoda | Insecta | Ecnomidae | *Ecnomus tenellus* | Etene |  |  | 2.13 |  |  |
| Arthropoda | Insecta | Ephemerellidae | *Ephemerella* sp. | Ephem |  |  |  |  | 1.33 |
| Arthropoda | Insecta | Ephemeridae | *Ephemera shengmi* | Eshen |  |  |  |  | 17.44 |
| Arthropoda | Insecta | Gomphidae | *Gomphus postocularis* | Gpost |  |  | 0.36 |  | 1.22 |
| Arthropoda | Insecta | Gomphidae | *Gomphus pryeri* | Gprye |  |  | 1.07 |  |  |
| Arthropoda | Insecta | Macromiidae | *Macromia amphigena* | Mamph |  |  | 3.2 |  |  |
| Arthropoda | Insecta | Notodontidae | *Phalera flavescens* | Pflav | 3.67 | 2.67 |  | 4.67 |  |
| Arthropoda | Insecta | Polycentropodidae | *Neureclipsis* sp. | Neure |  |  | 0.35 |  | 2.67 |
| Mollusca | Bivalvia | Unionidae | *Anodonta woodiana* | Awood |  |  | 1.42 |  |  |
| Mollusca | Bivalvia | Unionidae | *Unio douglasiae* | Udoug |  |  | 0.04 |  |  |
| Mollusca | Gastropoda | Lymnaeidae | *Galba truncatula* | Gtrun |  | 0.33 |  |  |  |
| Mollusca | Gastropoda | Lymnaeidae | *Radix ovata* | Rovat |  |  |  |  | 9.22 |
| Mollusca | Gastropoda | Semisulcospiridae | *Semisulcospira amurensis* | Samur |  |  | 0.04 |  |  |
| Mollusca | Gastropoda | Valvatidae | *Valvata piscinalis* | Vpisc |  |  | 3.2 |  | 0.67 |
| Mollusca | Gastropoda | Viviparidae | *Bellamya aeruginosa* | Baeru |  | 4 | 0.26 |  | 3.78 |
| Mollusca | Gastropoda | Viviparidae | *Bellamya purificata* | Bpuri |  |  | 0.21 |  |  |
| Mollusca | Gastropoda | Viviparidae | *Cipangopaludina chinensis* | Cchin |  |  |  |  | 7 |
| Mollusca | Gastropoda | Viviparidae | *Viviparus chui* | Vchui | 4.5 |  |  | 11 |  |
